# Percutaneous Iatrogenic Atrial Septal Defect Closure Post Transcatheter Mitral Valve Repair: Revisiting MITHRAS Trial in a Larger Cohort

**DOI:** 10.7759/cureus.106390

**Published:** 2026-04-03

**Authors:** Hammad S Chaudhry, Dawood Shehzad, Amr Moustafa, Muhammad Faisal Riaz, Logan Johnke, Sunia S Chaudhary, Dawlat Khan

**Affiliations:** 1 Internal Medicine, University of South Dakota Sanford School of Medicine, Sioux Falls, USA; 2 Internal Medicine, Rawalpindi Medical University, Rawalpindi, PAK; 3 Cardiology, Poonch Medical College, Rawalakot, PAK

**Keywords:** healthcare cost and utilization project (hcup), iatrogenic atrial septal defect, national inpatient sample (nis), percutaneous atrial septal defect closure, retrospective research, transcatheter mitral valve repair

## Abstract

Introduction

Transcatheter mitral valve repair (TMVR) creates an iatrogenic atrial septal defect (iASD) that may persist and affect hemodynamics. Although the MITHRAS (Closure of Iatrogenic Atrial Septal Defects Following Transcatheter Mitral Valve Repair) trial showed no benefit of routine closure, its small size and selective population limit generalizability, and observational data remain conflicting. We hypothesized that percutaneous iASD closure (PC-iASD) would be associated with differences in in-hospital clinical and hemodynamic outcomes compared with no closure.

Methods

Using the National Inpatient Sample (NIS), we evaluated national prevalence, baseline characteristics, and in-hospital outcomes of PC-iASD among patients undergoing TMVR from 2020 to 2022.

Results

We identified 4,965 weighted TMVR hospitalizations; 431 (8.7%) underwent PC‑iASD closure during the same admission. Compared with TMVR only, the closure cohort had a higher prevalence of pulmonary hypertension (245 (56.9%) vs. 1735 (40.5%), p = 0.0034) and heart failure (411 (95.4%) vs. 3767 (87.9%), p = 0.0396). There were no significant differences in chronic obstructive pulmonary disease, coronary artery disease, end-stage renal disease, or tricuspid regurgitation between the two groups. After multivariate adjustment, there was no difference in in‑hospital mortality, cardiogenic shock, acute heart failure, acute right heart failure, cardiac tamponade, or need for mechanical circulatory support among the two cohorts.

Conclusion

There have been conflicting results over numerous retrospective and prospective studies regarding the outcomes of post-TMVR iASD closure. Potential benefits of right-sided volume overload may be offset by higher left-atrial pressures after closure. The wide confidence (albeit nonsignificant) toward the development of acute right heart failure, pericardial effusion, and cardiac tamponade raises the possibility of better patient selection or some iASD closure procedural modifications that can help improve outcomes.

## Introduction

Transcatheter mitral valve repair (TMVR) has emerged as an alternative to surgery for severe mitral regurgitation in patients at high or prohibitive surgical risk. The procedure requires large-bore transseptal puncture (24 to 35 F), creating an iatrogenic atrial septal defect (iASD) that may persist [1]. Persistent iASDs have been linked to right ventricular (RV) volume overload, pulmonary hypertension (PH), and hypoxemia [2-4]. While some observational series suggest hemodynamic benefit from percutaneous iatrogenic atrial septal defect closure (PC-iASD), others report neutral or even deleterious effects related to left atrial (LA) pressure rise after closure [5-7].

The randomized MITHRAS (Closure of Iatrogenic Atrial Septal Defects Following Transcatheter Mitral Valve Repair) trial (Lurz et al., 2021) found no reduction in mortality or heart failure (HF) hospitalizations with routine closure following TMVR, but the study was small (n = 80) and excluded high-risk hemodynamic subgroups [1]. Subsequent registries and case series report conflicting outcomes for closure after TMVR, highlighting the need for large-scale analysis [8-12].

Given the prior conflicting data regarding the hemodynamic and clinical impact of persistent iASD, we hypothesized that PC-iASD following TMVR would be associated with a measurable difference in in-hospital mortality and hemodynamic outcomes in a real-world population. The present study leverages the large national administrative database, the NIS 2020-2022, to determine the national prevalence of PC-iASD following TMVR, compare baseline and procedural characteristics, and the primary outcomes, i.e., in-hospital mortality, and secondary outcomes, i.e., hemodynamic complications and need for mechanical support, with those who did not undergo PC-iASD post TMVR. We further aim to revisit MITHRAS in a “real-world” cohort with a broader patient population with a more diverse risk spectrum. The large patient population studied in this observational study will help clarify and contextualize the findings of previous literature and help inform future decisions regarding patient selection for PC-iASD intervention.

## Materials and methods

Data source

Data were extracted from the Healthcare Cost and Utilization Project (HCUP) National Inpatient Sample (NIS) for 2020-2022, a 20% stratified sample of the US hospital discharges weighted for national estimates [13].

Study population

Hospitalizations with the International Classification of Diseases, Tenth Revision (ICD-10) procedure codes for TMVR were included. Exclusions were age < 18 years, prior heart valve replacement, repair, and congenital atrial septal defect/patent foramen ovale. PC-iASD was identified by percutaneous closure codes during the index admission. Two groups were defined as TMVR + PC-iASD and TMVR only.

Covariates and outcomes

Variables included sex, pre-morbid conditions, which included prior coronary artery bypass surgery and prior pacemaker placement, and comorbidities, which included heart failure, right heart failure, systemic hypertension, pulmonary hypertension, atrial fibrillation/flutter, end-stage renal disease, tricuspid regurgitation, diabetes mellitus, coronary artery disease, and chronic obstructive pulmonary disease per Elixhauser definitions [14]. The univariate and multivariate outcomes reported are strictly in-hospital outcomes. The primary outcome was in‑hospital mortality. Secondary outcomes were cardiogenic shock, acute heart failure (HF), acute right‑heart failure (RHF), cardiac tamponade, use of mechanical circulatory support (MCS), respiratory failure, length of stay (LOS), and total hospital charges. Outcome variables except MCS were identified using the International Classification of Diseases, Tenth Revision, Clinical Modification (ICD-10-CM) diagnosis codes. MCS was defined using the International Classification of Diseases, 10th Revision, Procedure Coding System (ICD-10-PCS) procedure codes. A complete list of codes used to define exposures and outcomes is provided in the Supplementary Appendix.

Statistical analysis

Survey weights and variance estimates were applied. All analyses incorporated HCUP-provided discharge weights to generate national estimates, with appropriate stratification and clustering to account for the complex survey design. Categorical variables were compared using design-based Pearson chi-square tests with Rao-Scott design correction to account for complex survey design. Survey-weighted univariate and multivariable logistic regression adjusted for demographics and clinical covariates was performed. Statistical significance was assessed using design-adjusted Wald tests. Multivariable models were adjusted for patient sex, hospital characteristics, and clinically relevant comorbidities, including hypertension, diabetes mellitus, chronic kidney disease, coronary artery disease, heart failure, and other variables derived from the Elixhauser comorbidity index. Interaction terms were selected a priori based on clinical relevance and tested in multivariable models. Missing data were handled using complete-case analysis, consistent with HCUP recommendations. Analyses used Stata 18 (StataCorp LLC, College Station, TX).

Ethics

This analysis used publicly available, de‑identified HCUP‑NIS data and was exempt from institutional review board oversight.

## Results

Cohort and prevalence

Among 4,965 weighted TMVR hospitalizations, 431 (8.7%) underwent PC‑iASD during the same admission. This rate is four to five times higher than that seen after mitral transcatheter edge‑to‑edge repair (TEER), reflecting larger transseptal sheaths and longer procedural dwell time [4].

Baseline and procedural characteristics

Compared with the TMVR-only group, TMVR patients who underwent PC-iASD had a higher prevalence of pulmonary hypertension (245 (56.9%) vs. 1735 (40.5), p = 0.0034) and heart failure (411 (95.4%) vs. 3767 (87.9%), p = 0.0396). There was no significant difference in the prevalence of systemic hypertension, atrial fibrillation/flutter, end-stage renal disease (ESRD), chronic obstructive pulmonary disease (COPD), coronary artery disease (CAD), tricuspid regurgitation (TR), prior coronary artery bypass grafting (CABG), and prior pacemaker placement among the two cohorts (p > 0.05). Baseline characteristics are summarized in Table 1.

**Table 1:** Baseline and procedural characteristics of patients undergoing TMVR with or without PC-iASD. TMVR, transcatheter mitral valve repair; PC-iASD, percutaneous closure of iatrogenic atrial septal defect; CABG: coronary artery bypass grafting; COPD, chronic obstructive pulmonary disease; ESRD, end-stage renal disease. Statistical test: Rao-Scott adjusted chi-square test.

Characteristic	TMVR only	TMVR + PC-iASD	Statistical test value	p-value
Female sex (%)	54.0	51.2	0.31	0.577
Tricuspid regurgitation (%)	5.6	5.8	0.01	0.940
Prior CABG (%)	20.6	18.6	0.19	0.660
Prior permanent pacemaker (%)	14.1	17.4	0.75	0.387
Systemic hypertension (%)	85.0	80.2	1.33	0.249
Pulmonary hypertension (%)	40.5	56.9	8.60	0.003
Atrial fibrillation/flutter (%)	46.1	53.5	1.86	0.173
Coronary artery disease (%)	58.9	51.2	1.66	0.198
Heart failure (%)	88.0	95.4	4.24	0.039
Diabetes mellitus (%)	30.8	26.7	0.59	0.443
COPD (%)	17.4	20.9	0.64	0.424
ESRD (%)	6.3	8.1	0.48	0.489

Clinical outcomes

*Univariate Analysis*

Univariate analysis outcomes were as follows: in‑hospital mortality (OR: 1.69; 95% CI: 0.68-4.20; p = 0.260), cardiogenic shock (OR: 1.33, 95% CI: 0.63-2.78, p = 0.451), postoperative shock (OR: 0.93, 95% CI: 0.46-1.88, p = 0.846), acute heart failure (OR: 1.24, 95% CI: 0.73-2.11, p = 0.431), acute right heart failure (OR: 1.77, 95% CI: 0.20-15.28, p = 0.605), cardiac tamponade (OR: 1.06, 95% CI: 0.13-8.40, p = 0.959), pericardial effusion (OR: 2.37, 95% CI: 0.88-6.37, p = 0.086), mechanical circulatory support (OR: 1.12, 95% CI: 0.45-2.76, p = 0.808), and acute respiratory failure (OR: 0.80, 95% CI: 0.41-1.54, p = 0.499). Although crude outcomes appeared numerically worse in the PC-iASD cohort, none reached statistical significance, underscoring the strong baseline-risk gradient that drives the operator’s decision to close iASD. Univariate analysis outcomes are shown in Table 2.

**Table 2:** Univariate survey-weighted analysis of in-hospital outcomes associated with PC-iASD following TMVR. OR, odds ratio; TMVR, transcatheter mitral valve replacement; PC-iASD, percutaneous closure of iatrogenic atrial septal defect. Statistical test: Design-adjusted Wald F test.

Outcome	Effect estimate (OR) (TMVR + PC-iASD vs. TMVR only)	95% confidence interval	Statistical test value	p-value
In-hospital mortality	1.69	0.68-4.20	1.27	0.260
Cardiogenic shock	1.33	0.63-2.78	0.57	0.451
Postoperative shock	0.93	0.46-1.88	0.04	0.846
Acute heart failure	1.24	0.73-2.11	0.62	0.431
Acute right heart failure	1.77	0.20-15.28	0.27	0.605
Cardiac tamponade	1.06	0.13-8.40	0.00	0.959
Pericardial effusion	2.37	0.88-6.37	2.94	0.086
Mechanical circulatory support	1.12	0.45-2.76	0.06	0.808
Acute respiratory failure	0.80	0.41-1.54	0.46	0.499

Multivariate Analysis

After adjustment for demographics and comorbidities, PC‑iASD was not associated with higher odds of in‑hospital mortality (adjusted odds ratio (aOR): 1.81, 95% CI: 0.72-4.58, p = 0.209), cardiogenic shock (aOR: 1.10, 95% CI: 0.53-2.28, p = 0.792), postoperative shock (aOR: 0.77, 95% CI: 0.38-1.55, p = 0.466), acute heart failure (aOR: 1.02, 95% CI: 0.60-1.74, p = 0.939), acute right heart failure (aOR: 1.43, 95% CI: 0.17-11.81, p = 0.740), cardiac tamponade (aOR: 1.28. 95% CI: 0.15-10.88, p = 0.824), pericardial effusion (aOR: 2.36, 95% CI: 0.87-6.43, p = 0.092), mechanical circulatory support (aOR: 1.10, 95% CI: 0.45-2.69), p = 0.842), or acute respiratory failure (aOR: 0.73, 95% CI: 0.37-1.41, p = 0.344). Clinically relevant interaction terms were tested in multivariable models, and no statistically significant interactions were identified. Multivariate analysis outcomes are shown in Table 3.

**Table 3:** Multivariate survey-weighted analysis of in-hospital outcomes associated with PC-iASD following TMVR. aOR, adjusted odds ratio; TMVR, transcatheter mitral valve repair; PC-iASD, percutaneous closure of atrial septal defect. Statistical test: Design-adjusted Wald F test.

Outcome	Effect estimate (aOR) (TMVR + PC-iASD vs. TMVR only)	95% confidence interval	Statistical test value	p-value
In-hospital mortality	1.81	0.72-4.58	0.99	0.209
Cardiogenic shock	1.10	0.53-2.28	3.30	0.709
Postoperative shock	0.77	0.38-1.55	3.91	0.466
Acute heart failure	1.02	0.60-1.74	2.19	0.939
Acute right heart failure	1.43	0.17-11.81	1.45	0.740
Cardiac tamponade	1.28	0.15-10.88	3.08	0.824
Pericardial effusion	2.36	0.87-6.43	1.07	0.086
Mechanical circulatory support	1.10	0.45-2.69	1.30	0.842
Acute respiratory failure	0.73	0.37-1.41	2.91	0.344

**Figure 1:**
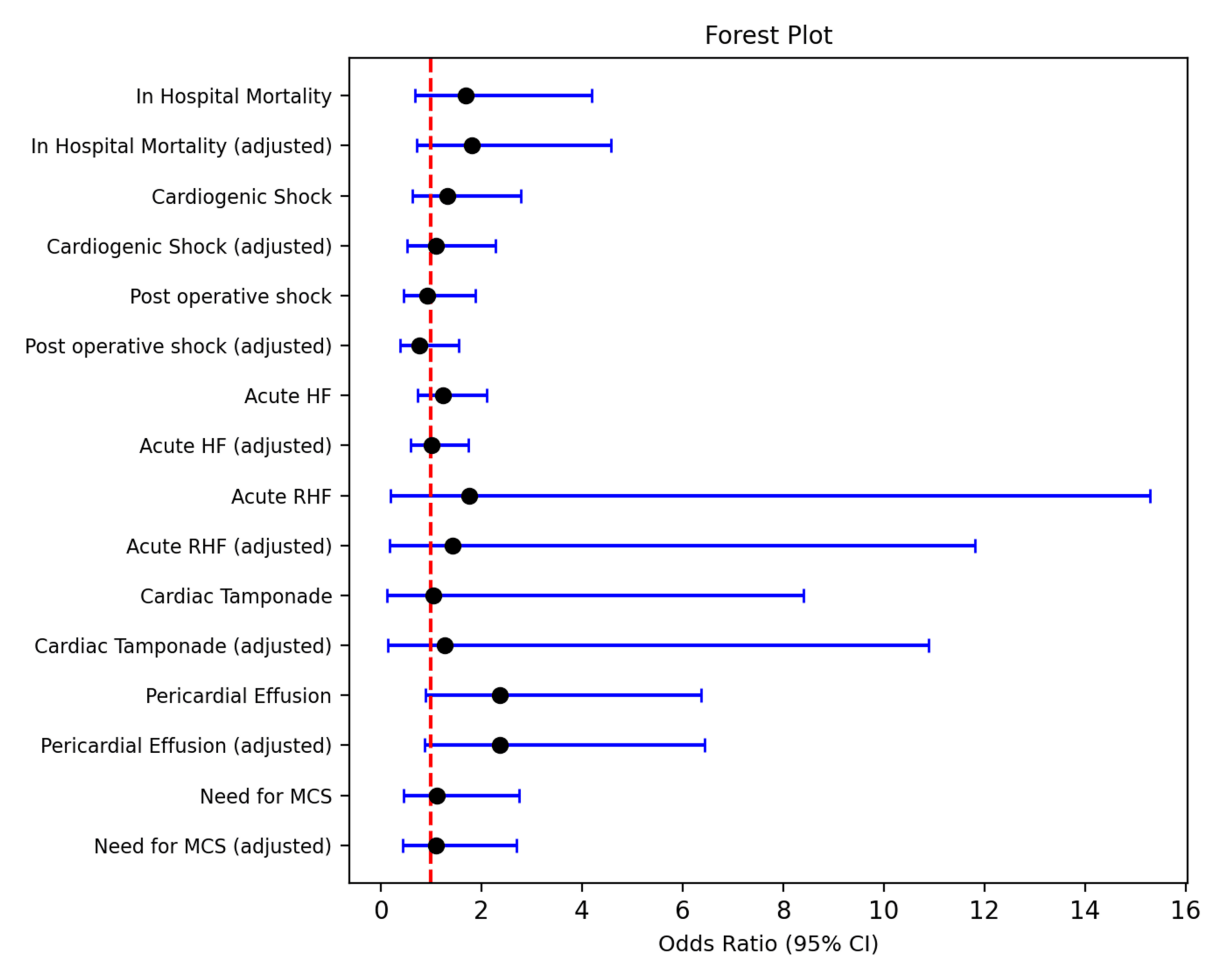
Forest plot demonstrating unadjusted and adjusted odds ratios for in-hospital outcomes among the two study cohorts. HF, heart failure; RHF, right heart failure; MCS, mechanical circulatory support.

Figure 1 depicts adjusted estimates with confidence intervals crossing unity.

Each estimate crosses unity; the red dashed vertical line at OR = 1.0 emphasizes the neutral effect. Confidence intervals are intentionally wide for rare outcomes such as acute RHF and tamponade, consistent with low event counts.

## Discussion

This national-level analysis demonstrates that ≈9% of TMVR patients undergo PC-iASD and that, after risk adjustment, there is no increase in mortality or major complications. These results expand upon MITHRAS, providing evidence in a five-year real-world cohort of nearly 5000 patients [1,2]. The observed increase in closure frequency reflects both operator comfort and the availability of dedicated occluder devices.

Persistent iASD after TMVR is common (30-60%), particularly with sheaths ≥ 32 F. Left‑to‑right shunting may reduce LA pressure at the expense of RV volume load; closure can raise LA pressure by 3-6 mmHg and potentially increase pulmonary venous hypertension. Thus, closure offers hemodynamic trade-offs [15]. Our data suggest that most operators select closure for patients already demonstrating right-sided strain or hypoxemia, rather than routine use. The neutral outcomes after adjustment imply that closure per se is safe when clinically indicated.

Several registries have reported similar findings. Hashem et al. (2023) analyzed > 900 combined TEER/TMVR cases and found no difference in 30-day mortality or HF readmission after closure [4]. Ranard et al. (2023) confirmed the absence of adverse impact in TMVR-specific cohorts [3]. Conversely, Morse et al. (2025) demonstrated that closure in patients with severe PH may acutely increase mean LA pressure by > 5 mmHg [16]. The present study, therefore, bridges MITHRAS (transcatheter edge-to-edge repair, low risk) and later TMVR series (high risk), showing consistent safety but no routine benefit [1,5].

Beyond PH and RHF, multivariable interaction testing suggests that older age, atrial fibrillation, and chronic kidney disease may contribute most to mortality risk, independent of closure status. These factors likely reflect overall frailty rather than a procedure-specific effect.

In light of the contemporary observational evidence, PC-iASD closure after TMVR does not seem to be routinely recommended. Closure should be considered in patients with hemodynamically significant right-to-left shunting or signs of increasing right-ventricular volume overload. A careful transesophageal echocardiogram (TEE) assessment, including defect size and pulmonary to systolic blood flow ratio (Qp:Qs), should guide the decision to close. In patients with pulmonary hypertension, staged closure or a fenestrated device may help reduce the risk of an acute rise in left-atrial pressure. Future guidelines should provide clearer direction on how to manage iASDs after transseptal valve procedures.

Prospective multicenter registries should incorporate real-time hemodynamic data and AI-based modeling to predict which patients benefit from closure. Integration of left-atrial compliance metrics and pulmonary vascular resistance could inform personalized algorithms. Emerging 3D computed tomography (CT) and TEE fusion may refine closure timing and device selection.

Strengths and limitations

The principal strength of this study is its large, nationally representative cohort reflecting real-world practice, enhancing generalizability beyond prior smaller studies. However, limitations inherent to administrative data include potential coding inaccuracies and a lack of granular detail, particularly echocardiographic parameters and post-discharge outcomes. Importantly, key physiologic variables such as Qp:Qs and pulmonary vascular resistance were unavailable, limiting mechanical insight. Residual confounding due to indication bias is likely, as closure was more frequently performed in higher-risk patients, which may not be fully accounted for despite adjustment. Additionally, restriction to in-hospital outcomes precludes assessment of long-term effects. These factors may partly explain the observed neutral association after adjustment, as selective use in higher-risk patients and unmeasured confounding could bias results toward the null. As such, the findings should be interpreted as supportive of procedural safety but hypothesis-generating rather than definitive.

## Conclusions

Our findings support the procedural safety of iASD closure post TMVR. However, in light of findings from prior literature, there seems to be no evidence of any benefit from routine closure of iASD after TMVR. Rather, closure appears to be warranted only in select patients with clear clinical or hemodynamic indications, such as a right-sided strain or hypoxemia. Conclusively, a patient-specific physiology-guided approach, particularly in those with pulmonary hypertension or right-ventricular dysfunction, should guide decision-making for PC-iASD post TMVR.

## Appendices

Supplementary appendix: ICD-10 codes used in the study

**Table 4:** ICD-10 codes for principle variables. ICD-10, International Classification of Diseases, Tenth Revision; PCS, Procedure Coding System.

Variable	ICD-10 category	Code
Transcatheter mitral valve replacement (TMVR)	ICD-10-PCS	02RG3
Percutaneous atrial septal defect closure (PC-iASD)	ICD-10-PCS	02U53, 02U54

**Table 5:** ICD-10 codes for exclusion variables. ICD-10, International Classification of Diseases, Tenth Revision; PCS, Procedure Coding System; CM, Clinical Modification.

Variable	ICD-10 category	Code
Prior heart valve replacement or repair	ICD-10-PCS	02RF37H, 02RF37Z, 02RF38H, 02RF38Z, 02RF3JH, 02RF3JZ, 02RF3KZ 02RF07, 02RF07Z, 02RF08, 02RF08Z, 02RF0JZ, 02RF0KZ 02UJ07Z, 02UJ08Z, 02UJ0JZ, 02UJ0KZ, 02QJ0ZG, 02QJ0ZZ 02RJ07Z, 02RJ0JZ 02UG3JZ
Congenital atrial septal defect (ASD)	ICD-10-CM	Q2110

**Table 6:** ICD-10 codes for baseline variables. ICD-10, International Classification of Diseases, Tenth Revision; PCS, Procedure Coding System; CM, Clinical Modification; CABG, coronary artery bypass grafting.

Variable	ICD-10 category	Code
Tricuspid regurgitation	ICD-10-CM	I361, I362
Prior CABG	ICD-10-CM	0210088, 0210089, 021008C, 021008F, 0210093, 0210098, 0210099, 021009C, 021009F, 02100A3, 02100A8, 02100A9, 02100AC, 02100AF, 02100J3, 02100J8, 02100J9, 02100JC, 02100JF, 02100K3, 02100K8, 02100K9, 02100KC, 02100KF, 02100Z3, 02100Z8, 02100Z9, 02100ZC, 02100ZF, 021008W, 021009W, 02100AW, 02100JW, 02100KW, 0210344, 02103D4, 0210444, 02104D4, 0210488, 0210489, 021048C, 021048F, 0210493, 0210498, 0210499, 021049C, 02104A3, 02104A8, 02104J9, 02104JC, 02104JF, 021104K3, 02104K8, 02104K9, 02104KC, 02104KF, 02104Z3, 02104Z8, 02104Z9, 02104ZC, 021048W, 021049W, 021049F, 02104AW, 02104JW, 02104KW, 02104ZF, 0211344, 02113D4, 0211444, 02114D4, 021108W, 021109W, 02110AW, 02110JW, 02110KW, 021148W, 021149W, 02114AW, 02114JW, 02114KW 0212344, 02123D4, 0212444, 02124D4, 021208W, 021209W, 02120AW, 02120JW, 02120KW, 021248W, 021249W, 02124AW, 02124JW, 02124KW, 0213344, 02133D4, 0213444, 02134D4, 021308S, 021309W, 02130AW, 02130JW, 02130KW, 021348W, 021349W, 02134AW, 02134JW, 02134KW, 021K0Z8, 021K0Z9, 021K0ZC, 021K0ZW, 021K0Z5 021K4Z8, 021K4Z9, 021K4ZC, 021K4ZW, 021K4Z5, 021L0Z8, 021L0Z9, 021L0ZC, 021L0Z5, 021L4Z8, 021L4Z9, 021L4ZC, 021L4Z5, 02QA4ZZ, 02QA3ZZ, 02QA0ZZ 02QB4ZZ, 02QB3ZZ, 02QB0ZZ, 02QC4ZZ, 02QC3ZZ, 02QC0ZZ
Prior permanent pacemaker (PPM)	ICD-10-PCS	0JH604, 0JH605, 0JH606Z, 0JH607, 0JH608, 0JH609, 02H63JZ, 02HK3JZ, 02HK3NZ
Systemic hypertension	ICD-10-CM	I10, I11, I12, I13, I15, I16, I1A
Pulmonary hypertension	ICD-10-CM	I27
Atrial fibrillation/flutter	ICD-10-CM	I480, I4811, I4819, I482, I483, I484, I489, I4892
Coronary artery disease	ICD-10-CM	I25.10, I25.11, I25.111, I25.118, I25.119, I25.2, I25.3, I25.4, I25.41, I25.42, I25.5, I25.6, I25.7, I25.70, I25.700, I25.701, I25.708, I25.709, I25.71, I25.710, I25.711, I25.718, I25.719, I25.72, I25.720, I25.721, I25.728, I25.729, I25.73, I25.730, I25.731, I25.738, I25.739, I25.75, I25.750, I25.751, I25.758, I25.759, I25.76, I25.760, I25.761, I25.768, I25.769, I25.79, I25.790, I25.791, I25.798, I25.799, I25.8, I25.81, I25.810, I25.811, I25.812, I25.82, I25.83, I25.84, I25.89, I25.9 Z95.1
Heart failure	ICD-10-CM	I500, I501, I502, I5020, I5021, I5022, I5023, I503, I5030, I5031, I5032, I5033, I504, I5040, I5041, I5042, I5043, I509, I5081, I50810, I50811, I50812, I50813, I50814, I5082, I5083, I5084, I5089, I509, I110, I1255, I130, I132, I0981, I099, I420, I425, I429, I43
Diabetes mellitus	ICD-10-CM	E10, E11, E12, E13, E14
Chronic obstructive pulmonary disease (COPD)	ICD-10-CM	J440, J441, J448, J449
End-stage renal disease (ESRD)	ICD-10-CM, ICD-10-PCS	N186, Z992, 5A1D, 5A1D7, 5A1D70, 5A1D70Z, 5A1D8, 5A1D80, 5A1D80Z, 5A1D9, 5A1D90, 5A1D90Z

**Table 7:** ICD-10 codes for outcome variables. ICD-10, International Classification of Diseases, Tenth Revision; PCS, Procedure Coding System; CM, Clinical Modification.

Variable	ICD-10 category	Code
Cardiogenic shock	ICD-10-CM	R570
Postoperative shock	ICD-10-CM	T811, T8110XA, T8119XA, R55, R570, R571, R579, T782, I971
Acute heart failure	ICD-10-CM	I5021, I5023, I5031, I5033, I5041, I5043
Acute right heart failure	ICD-10-CM	I50811, I50813
Cardiac tamponade	ICD-10-CM	I314
Pericardial effusion	ICD-10-CM	I313, I3139
Mechanical circulatory support (MCS)	ICD-10-PCS	5A0211D, 5A02210, 5A0221D, 5A15A2F, 5A1522F, 5A15A2G, 5A1522G, 5A15A2H, 5A1522H, 5A1523, 02HA3RJ, 02HA3RS, 02HA3RZ
Acute respiratory failure	ICD-10-CM	J9620, KK9621, J9622, J80, J9600, J9601, J9602, J9690, J9691, J9692
